# Supplementary material for: Harnessing Transcriptomic Signals for Amyotrophic Lateral Sclerosis to Identify Novel Drugs and Enhance Risk Prediction
Source: medRxiv. 2023 Jan 24:2023.01.18.23284589. Preprint. [Version 2] doi: 10.1101/2023.01.18.23284589 (PMC9901068; doi:10.1101/2023.01.18.23284589)
Supplement: Supplement 2 [file NIHPP2023.01.18.23284589v2-supplement-2.pdf]

medRxiv preprint doi: https://doi.org/10.1101/2023.01.18.23284589; this version posted January 24, 2023. The copyright holder for this preprint (which was not certified by peer review) is the author/funder, who has granted medRxiv a license to display the preprint in perpetuity.
It is made available under a CC-BY 4.0 International license .

Supplementary Information

1. GWAS quality control

GWAS summary statistics underwent quality control to extract variants present in the 1000 Genomes (1KG) phase 3 reference sample (1000 Genomes Project Consortium, 2015), remove ambiguous variants, remove variants with missing data, flip variants to match the reference, retain variants with a minor allele frequency (MAF) > 0.01 in the European subset of 1KG Phase 3, retain variants with a MAF > 0.01 in the GWAS sample, remove variants with a discordant MAF (> 0.2) between the reference and GWAS sample, remove variants with association *p*-values >1 or ≤ 0, remove duplicate variants, and remove variants with sample size > 3SD from the median sample size.

2. Fine-mapping

Independent genome-wide significant loci were defined using LD-based clumping ($r^2 < 0.1$, 500kb window), applying SuSiE to all variants within 500kb of each lead variant.

Fine-mapping is particularly sensitive to LD mismatch between GWAS summary statistics and the LD reference. Given we did not have estimates of LD from the original samples in the ALS GWAS, we set the number of causal signals within each locus to 1 (i.e., L = 1), as when L = 1 fine-mapping does not consider LD estimates at all and is therefore more robust. The limitation of the L = 1 assumption is that fine-mapping will be less powerful in loci where multiple causal signals are present.

As a sensitivity analysis, we ran fine-mapping using the default L = 10 parameter, allowing up to 10 independent causal signals within each locus, using the European ancestry subset of the 1KG reference to calculate LD.

3. KCL Brain Bank

The KCL Brain Bank dataset previously underwent quality control and standard eQTL analysis (Iacoangeli et al., 2021; Jones et al., 2021). We used a broader version of the dataset to previous publications without age and sex matching between individuals with and without ALS diagnosis as we are not trying to identify associations with ALS. Using GenoPredPipe (https://github.com/opain/GenoPred/tree/master/GenoPredPipe) and the 1KG Phase 3 reference, we identified individuals in KCL Brain Bank of European ancestry and calculated 10 genetic principal components. The final dataset consisted of 153 individuals, including 103 individuals diagnosed with ALS and 50 controls. The expression data was controlled for ALS status, gender, age, post-mortem delay, RIN (RNA integrity), surrogate variables and genetic principal components. We used only HapMap3 variants when generating TWAS weights to improve overlap with external datasets. The weights were created using FUSION software, implemented using a publicly available pipeline (https://github.com/opain/Calculating-FUSION-TWAS-weights-pipeline).

Post-mortem tissue samples from King's College London were collected under the ethical approval of the MRC London Neurodegenerative Diseases Brain Bank and under the regulations of the Human Tissue Act UK 2014. All post-mortem tissue was donated to the MRC London Neurodegenerative Diseases Brain Bank under standard ethical and Human Tissue Act procedures, with informed consent provided by the next of kin. Data generated from this material were anonymized and

medRxiv preprint doi: https://doi.org/10.1101/2023.01.18.23284589; this version posted January 24, 2023. The copyright holder for this preprint (which was not certified by peer review) is the author/funder, who has granted medRxiv a license to display the preprint in perpetuity.
It is made available under a CC-BY 4.0 International license .

analysed on a high-performance computing cloud (https://www.maudsleybrc.nihr.ac.uk/facilities/rosalind/) with data protection protocols in accordance with Department of Health Policy (UK) and the security standards set by the National Data Guardian. Ethical approval to process and analyse post-mortem samples stored at King's College London was provided by a local ethics committee at the Institute of Psychiatry, Psychology & Neuroscience, King's College London, and the MRC London Neurodegenerative Diseases Brain Bank.

4. FUSION/SMR

FUSION software implements an approach often referred to as TWAS, which infers differential expression/protein levels associated with the GWAS phenotype using multi-variant models predicting expression or protein levels (Gusev et al., 2016). These multi-variant models are not available for all eQTL datasets, partly because deriving these multi-variant models currently requires individual-level gene expression and genotype data.

SMR also infers differential expression/protein levels associated with the GWAS phenotype (Zhu et al., 2016), but aims to provide evidence for causal role of a given SNP on a trait mediated through gene expression. SMR only considers the genetic variant most strongly predicting expression or protein levels, thereby explaining less variance in expression/protein levels than FUSION multi-SNP models for genes which have secondary eQTL/pQTL effects. A current advantage of SMR over FUSION is that it can be applied using only eQTL summary statistics which are more widely available.

Both FUSION and SMR include an analysis to determine whether the overlapping genetic association for the phenotype and the gene expression is driven by the same causal variant (pleiotropy) or whether different causal variants that are in LD are driving the associations (linkage). FUSION uses the coloc package to perform Bayesian colocalisation (Giambartolomei et al., 2014). SMR uses the frequentist HEIDI test.

5. Drug enrichment methods

MAGMA gene-set enrichment analysis is based on MAGMA estimated gene associations and binary drug-gene interaction data (de Leeuw et al., 2015). This approach does not consider the direction of ALS-gene or drug-gene associations, so it identifies drugs that interact with genes enriched for association with ALS but does not indicate whether enriched drugs will decrease risk of ALS. MAGMA gene set enrichment analysis estimates the non-independence of gene associations by using an LD-based correlation matrix and a generalised least square model.

Gene co-regulation score (GCSC) regression is a method that leverages gene co-regulation to test for and enrichment of TWAS associations within gene-sets or associated with gene-properties (Siewert-Rocks et al., 2022). Like MAGMA, GCSC does not consider the direction of effect between the gene and the phenotype. GCSC was run using default settings and the publicly available co-regulation matrices, based on GTEx v7 expression (https://github.com/ksiewert/GCSC). We restricted the analysis to GTEx brain tissues and GTEx whole blood, consistent with gene expression panels included in our TWAS analysis. As currently required for use of the GCSC coregulation matrices, we performed a TWAS using the GTEx v7 expression panels as input for the GCSC analysis.

TWAS-based gene-set enrichment analysis (TWAS-GSEA) is based on TWAS estimated gene association and directional drug-gene interaction data (Pain et al., 2019). This approach does

medRxiv preprint doi: https://doi.org/10.1101/2023.01.18.23284589; this version posted January 24, 2023. The copyright holder for this preprint (which was not certified by peer review) is the author/funder, who has granted medRxiv a license to display the preprint in perpetuity.
It is made available under a CC-BY 4.0 International license .

consider the direction of ALS-gene and drug-gene associations, so enriched drugs using this method are suggested to induce expression changes reducing risk of ALS. TWAS-GSEA estimates the non-independence of gene associations using a predicted expression correlation matrix and a linear mixed model, using the lme4qtl R package (Ziyatdinov et al., 2018).

These three approaches have different advantages and limitations and are therefore complimentary. MAGMA gene associations have no clear mechanistic link to the phenotype and the enrichment analysis does not consider direction of effect. However, MAGMA will generally test for enrichment across more genes that TWAS-based enrichment as it considers ALS-associated genes acting via any mechanism (not only differential expression) and is not dependent on external eQTL datasets which are often limited in sample size thereby reducing coverage of the genome. GCSC has the advantage of pooling expression associations across expression panels, thereby improving coverage and statistical power to detect enrichment. TWAS-GSEA does not pool information across expression panels but does allow for the direction of effect in the TWAS to be considered, and is thereby able to highlight drugs that consistently reduce risk-associated differential expression.

6. Gene discovery results

SNP-based fine-mapping results assuming a single causal signal (L = 1) are summarised in Table S1. LD-based clumping identified 16 independent genome-wide significant associations, which were carried forward for fine-mapping analysis centred on the lead variant +/- 500kb. Within three loci a single variant was present in the 95% credible set. Within four loci the 95% credible set was contained within a given gene, with one 95% credible set within two overlapping genes. SNP-based fine-mapping results allowing for up to 10 causal signals (L = 10) are shown in Table S2, indicating the presence of multiple causal signals underlying genome-wide significant locus on chromosome 9, with 95% credible sets contained within the *C9orf72* and *MOB3B* genes.

TWAS using FUSION identified 197 FDR-significant and colocalised associations for ALS, including 101 unique genes (Table S3). TWAS using SMR identified 44 FDR significant associations passing the HEIDI test (indicating colocalisation), including 29 unique genes (Table S4). Across FUSION and SMR, 108 unique genes were identified as significant and colocalised, of which 22 were found by both TWAS and SMR.

PWAS using FUSION with ROSMAP and Banner pQTL data identified 8 FDR-significant and colocalised associations, including 6 unique genes, and thereby 2 were significant and colocalised according to both ROSMAP and Banner (Table S5). SMR using ROSMAP pQTL data identified 4 significant and colocalised associations, of which 3 were in common with FUSION PWAS results (Table S6). Of the 7 unique genes implicated using PWAS analysis, 3 were in common with TWAS results.

Across SNP fine-mapping, TWAS and PWAS analyses, 117 unique genes were identified as high-confidence associations.

medRxiv preprint doi: https://doi.org/10.1101/2023.01.18.23284589; this version posted January 24, 2023. The copyright holder for this preprint (which was not certified by peer review) is the author/funder, who has granted medRxiv a license to display the preprint in perpetuity.
It is made available under a CC-BY 4.0 International license .

Supplementary Figures

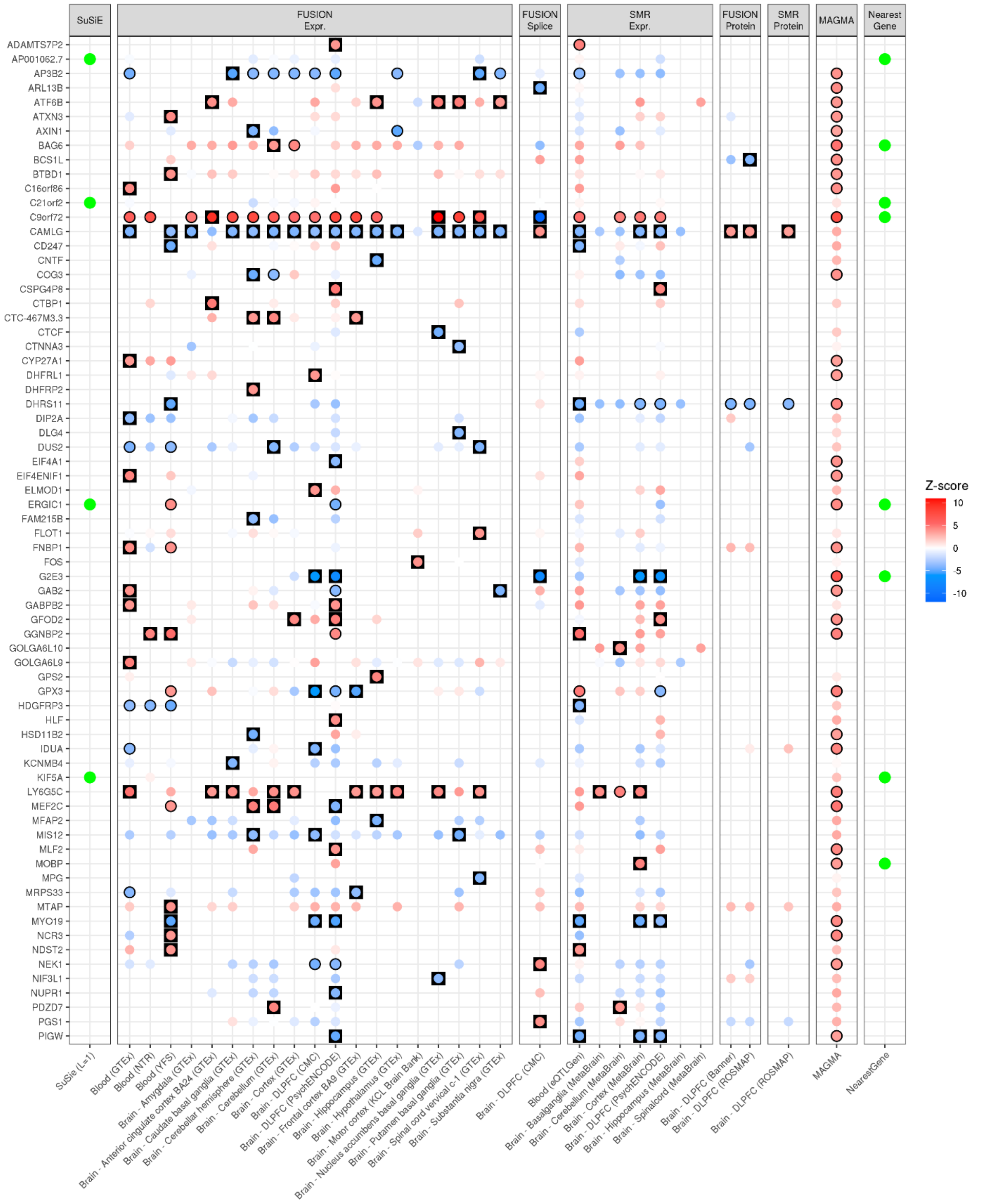

Figure S1a. High-confidence gene list for ALS defined using results of SNP fine-mapping, TWAS and PWAS. MAGMA and NearestGene results included for comparison. Results are separated by the method and external data used. Genes containing the 95% credible set from SNP fine-mapping (L = 1) are indicated using a green box. FUSION and SMR results are shown for all panels, with each boxed coloured according to the Z-score of association. Red indicates an increased expression/protein level in people diagnosed with ALS, and blue indicates decreased expression/protein level in people diagnosed with ALS. FUSION and SMR results have a black outline if the association was FDR significant, and are in a black square if the association was FDR significant and showed evidence of colocalisation. MAGMA associations are also shaded according to Z-score, although MAGMA cannot infer the direction of the effect, with FDR significant genes outlined in black. Genes nearest to lead variants within genome-wide significant loci are indicated using a green point.

medRxiv preprint doi: https://doi.org/10.1101/2023.01.18.23284589; this version posted January 24, 2023. The copyright holder for this preprint (which was not certified by peer review) is the author/funder, who has granted medRxiv a license to display the preprint in perpetuity.
It is made available under a CC-BY 4.0 International license .

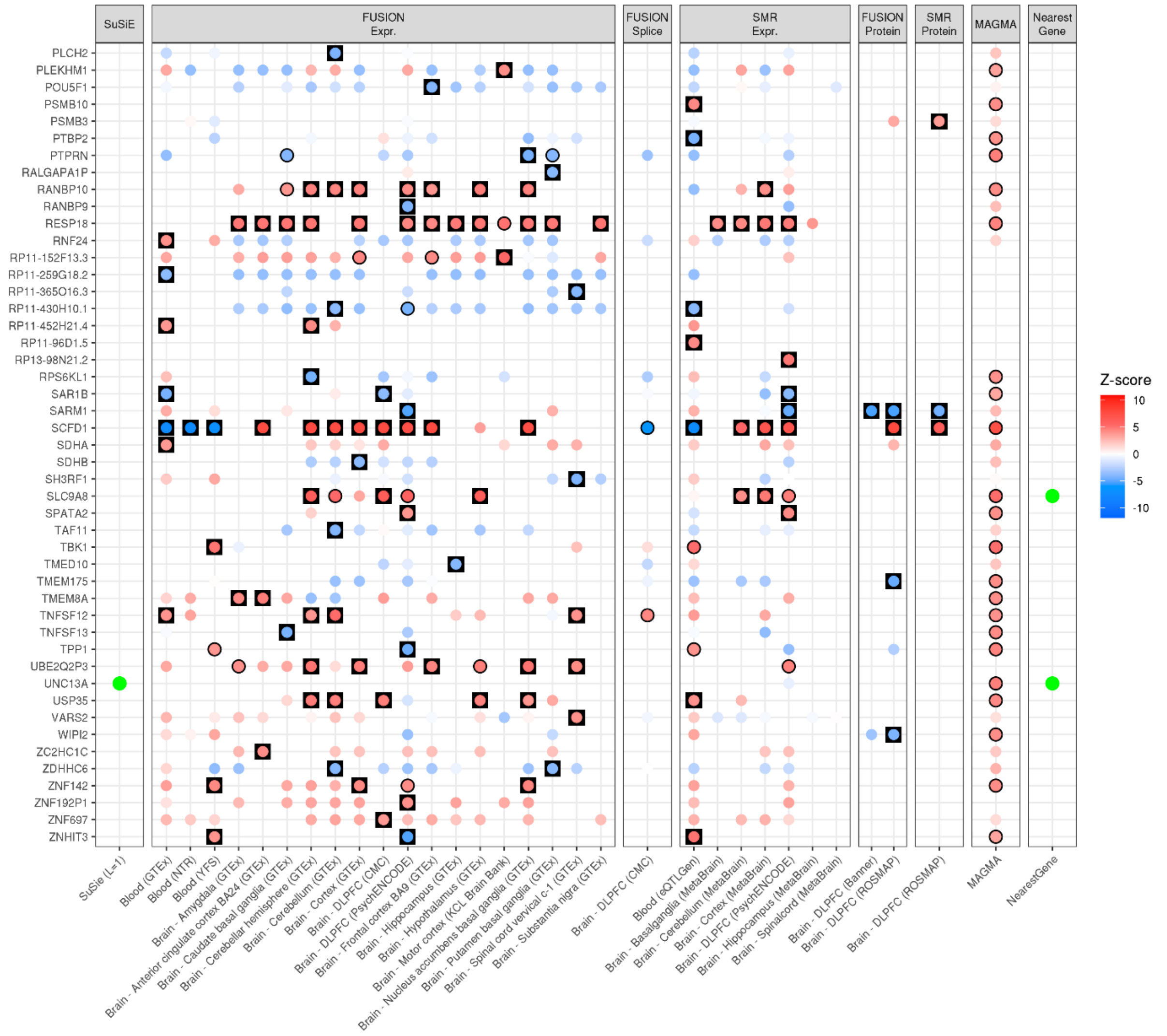

Figure S1b. High-confidence gene list for ALS defined using results of SNP fine-mapping, TWAS and PWAS. MAGMA and NearestGene results included for comparison. Results are separated by the method and external data used. Genes containing the 95% credible set from SNP fine-mapping (L = 1) are indicated using a green box. FUSION and SMR results are shown for all panels, with each boxed coloured according to the Z-score of association. Red indicates an increased expression/protein level in people diagnosed with ALS, and blue indicates decreased expression/protein level in people diagnosed with ALS. FUSION and SMR results have a black outline if the association was FDR significant, and are in a black square if the association was FDR significant and showed evidence of colocalisation. MAGMA associations are also shaded according to Z-score, although MAGMA cannot infer the direction of the effect, with FDR significant genes outlined in black. Genes nearest to lead variants within genome-wide significant loci are indicated using a green point.

medRxiv preprint doi: https://doi.org/10.1101/2023.01.18.23284589; this version posted January 24, 2023. The copyright holder for this preprint (which was not certified by peer review) is the author/funder, who has granted medRxiv a license to display the preprint in perpetuity.
It is made available under a CC-BY 4.0 International license .

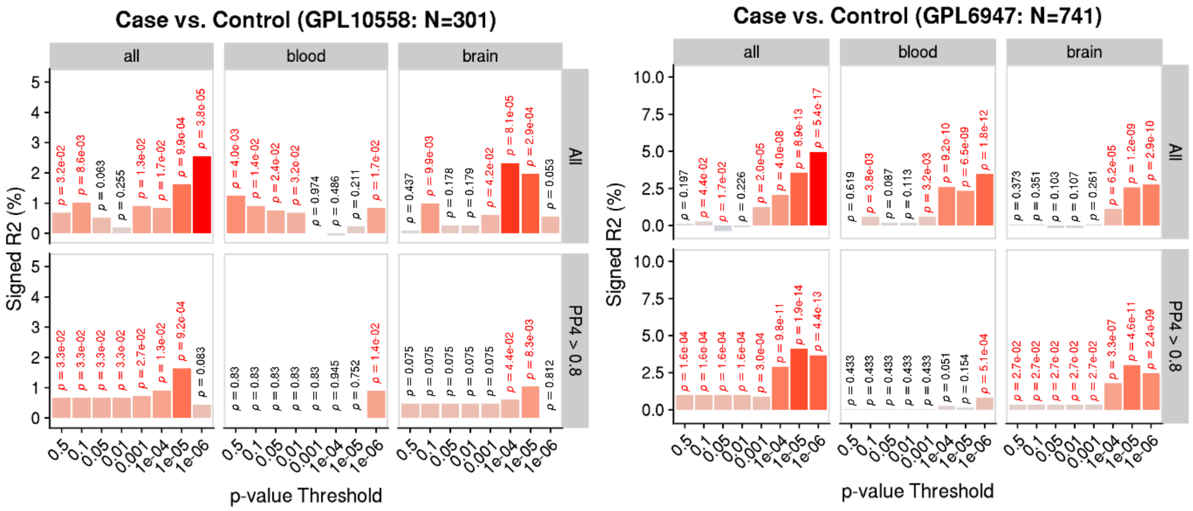

Figure S2. PTS association with ALS cases vs. controls split by target expression platform. Y-axis shows the variance explained on the liability scale assuming a prevalence of 1/300, signed by the direction of association. P-values are shown above each bar, with nominally significant associations highlighted in red. Results are shown when deriving PTS using all TWAS panels, only blood panels and only brain panels. Results are also shown when deriving PTS using only TWAS associations that showed evidence of colocalisation (PP4 > 0.8).

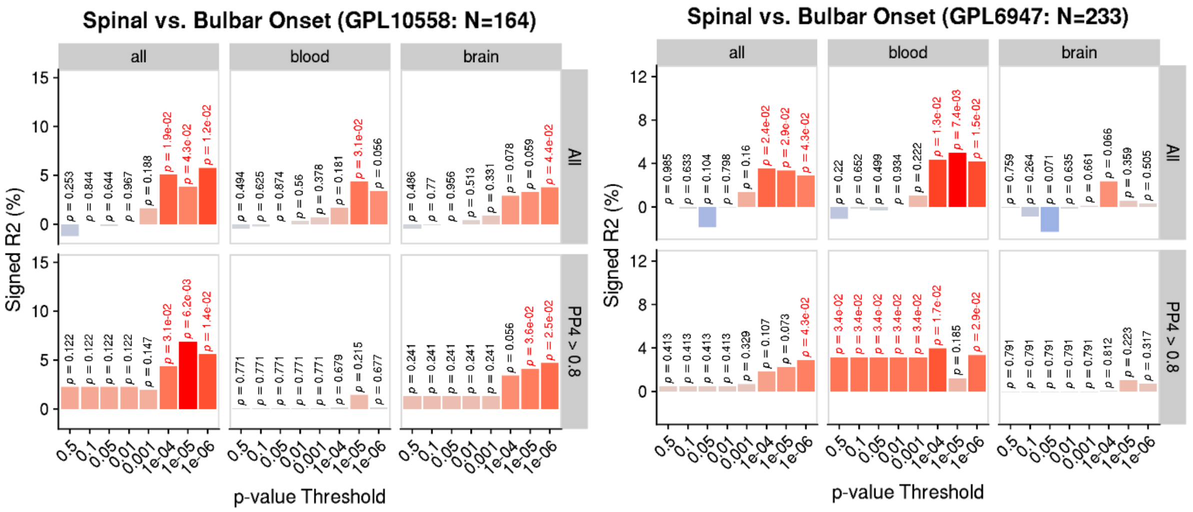

Figure S3. PTS association with site of onset in people with ALS split by target expression platform. Y-axis shows the variance explained on the liability scale assuming a prevalence of 50%, signed by the direction of association. P-values are shown above each bar, with nominally significant associations highlighted in red. Results are shown when deriving PTS using all TWAS panels, only blood panels and only brain panels. Results are also shown when deriving PTS using only TWAS associations that showed evidence of colocalisation (PP4 > 0.8).

medRxiv preprint doi: https://doi.org/10.1101/2023.01.18.23284589; this version posted January 24, 2023. The copyright holder for this preprint (which was not certified by peer review) is the author/funder, who has granted medRxiv a license to display the preprint in perpetuity.
It is made available under a CC-BY 4.0 International license .

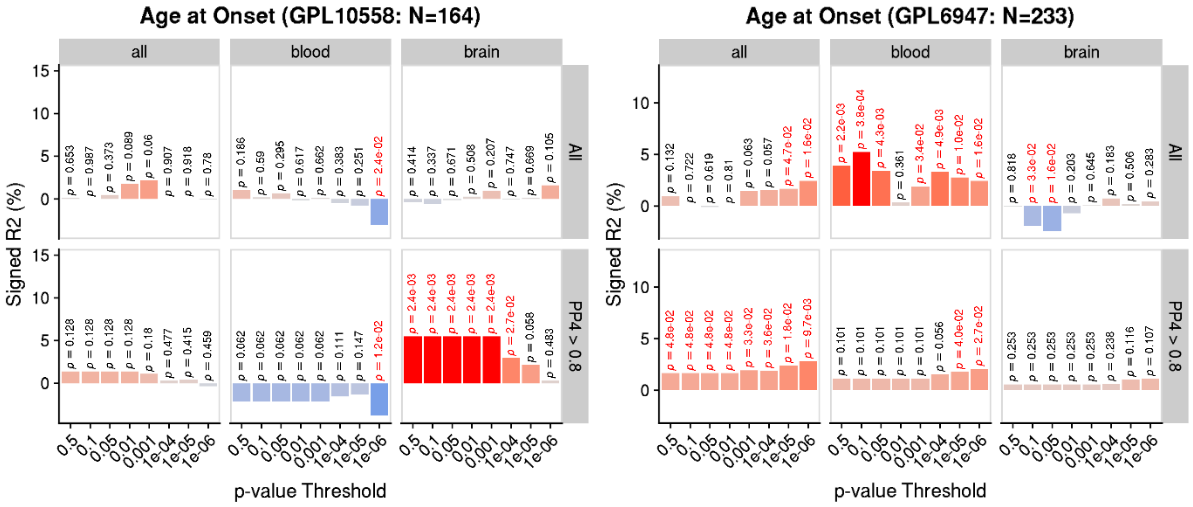

Figure S4. PTS association with age of onset in people with ALS split by target expression platform. Y-axis shows the variance explained on the observed scale, signed by the direction of association. P-values are shown above each bar, with nominally significant associations highlighted in red. Results are shown when deriving PTS using all TWAS panels, only blood panels and only brain panels. Results are also shown when deriving PTS using only TWAS associations that showed evidence of colocalisation (PP4 > 0.8).

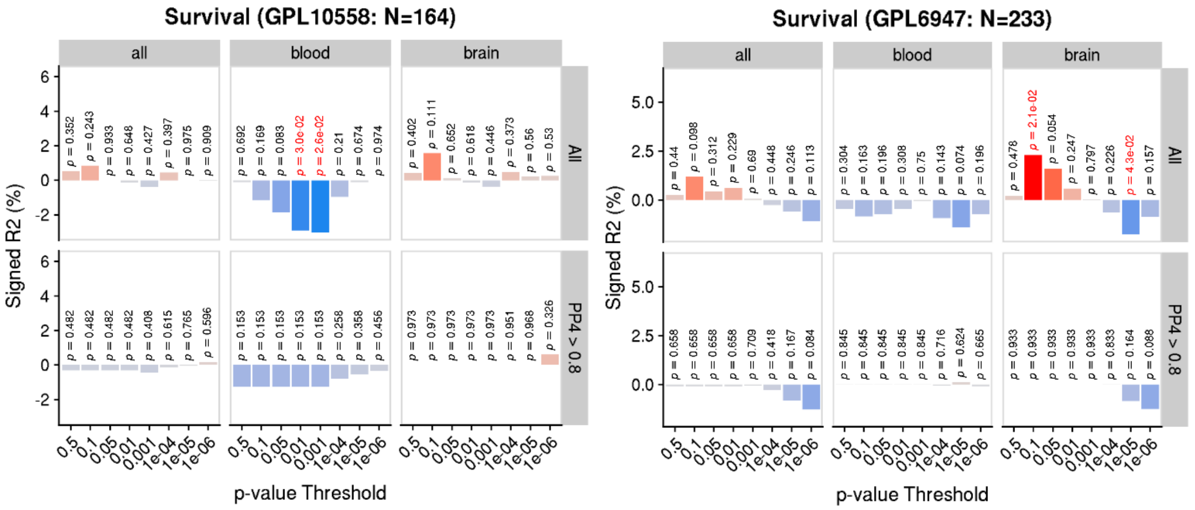

Figure S5. PTS association with site of onset in people with ALS split by target expression platform. Y-axis shows the variance explained on the oserved scale, signed by the direction of association. P-values are shown above each bar, with nominally significant associations highlighted in red. Results are shown when deriving PTS using all TWAS panels, only blood panels and only brain panels. Results are also shown when deriving PTS using only TWAS associations that showed evidence of colocalisation (PP4 > 0.8).

medRxiv preprint doi: https://doi.org/10.1101/2023.01.18.23284589; this version posted January 24, 2023. The copyright holder for this preprint (which was not certified by peer review) is the author/funder, who has granted medRxiv a license to display the preprint in perpetuity.
It is made available under a CC-BY 4.0 International license .

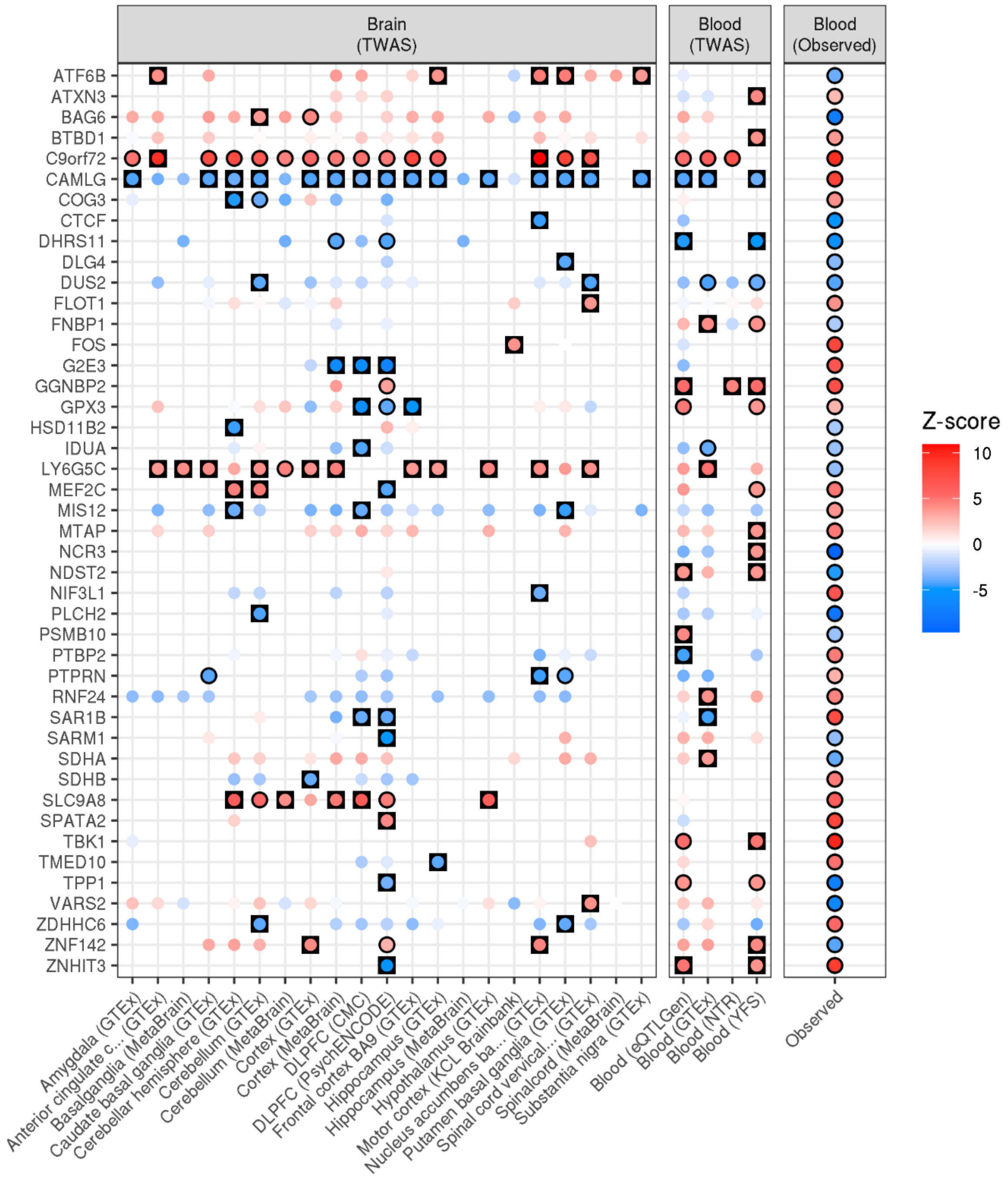

Figure S6. Comparison of observed and predicted (TWAS) differential expression associated with ALS risk. Showing only genes that were identified as high-confidence using TWAS (FUSION or SMR) and FDR significant observed differential expression. Results have a black outline if the association was FDR significant. TWAS results are highlighted in a black square if the association was FDR significant and showed evidence of colocalisation.
